# The Causal Role of Right Frontopolar Cortex in Moral Judgment, Negative Emotion Induction, and Executive Control

**DOI:** 10.32598/bcn.9.10.225

**Published:** 2019-01-01

**Authors:** Maryam Ziaei, Mansoureh Togha, Elham Rahimian, Jonas Persson

**Affiliations:** 1. Centre for Advanced Imaging, University of Queensland, Brisbane, Australia.; 2. School of Psychology, The University of Queensland, Brisbane, Australia.; 3. Department of Headache, Iranian Center of Neurological Research, Neuroscience Institute, Tehran University of Medical Sciences, Tehran, Iran.; 4. Haghighat and Shefa MRI Centers, Tehran, Iran.; 5. Aging Research Center (ARC), Karolinska Institute and Stockholm University, Stockholm, Sweden.

**Keywords:** Emotion induction, Frontopolar cortex, Personal impersonal Moral judgement, Executive control, Wisconsin Card Sorting Test

## Abstract

**Introduction:**

Converging evidence suggests that both emotional and cognitive processes are critically involved in moral judgment, and may be mediated by discrete parts of the prefrontal cortex. The current study aimed at investigating the mediatory effect of right Frontopolar Cortex (rFPC) on the way that emotions affect moral judgments.

**Methods:**

Six adult patients affected by rFPC and 10 healthy controls were included in the study. Participants made judgements on moral dilemmas after being shown either neutral or emotional pictures. The role of rFPC in executive control and emotional experience was also examined.

**Results:**

The study results showed that inducing an emotional state increased the number of utilitarian responses both in the patients and controls. However, no significant differences were observed between the patients and controls in response time or the number of utilitarian responses. Also, no significant differences were observed in personal and impersonal dilemmas before and after the emotion induction in intergroup comparisons. Results of the executive control tasks showed reduced performance in patients affected by rFPC compared with the controls.

**Conclusion:**

The results of the current study suggested that rFPC might not have a direct role in mediating emotional processes during moral judgments, but possibly this region is important in a network supporting executive control functions.

## Highlights

- Utilitarian responses increased following negative emotion induction in both healthy and Right frontopolar cortex (rFPC) damaged groups.
- rFPC may not have a role in mediating emotional processes during moral judgment.
- No difference between two groups was found in moral judgment.
- Feeling of guilt is affected by damage to rFPC.
- rFPC has specific role in inhibitory control and set shifting.

## Plain Language Summary

This study investigated the mediatory effect of right frontopolar cortex (rFPC) on moral judgments. Two groups of healthy controls and patients (with damage to their rFPCs) were tested with a moral judgment task. Negative emotion induction was presented prior to the task. While there were no difference between two groups in moral judgements and the effect of emotion induction, more utilitarian responses were found after negative emotion induction. Furthermore, the patients group performed worse on executive tasks tapping inhibitory control and reported more feeling of guilt after negative emotion induction. These findings highlight the important role of emotion on higher-order tasks, such as moral judgment, and the importance of rFPC in executive functioning and feeling of guilt.

## 1. Introduction

Moral judgement is commonly defined as evaluative judgements of the appropriateness of individuals’ behavior in the context of socialized awareness of right and wrong (Moll, Zahn, de Oliveira-Souza, Krueger, & Grafman, 2005). It is suggested that moral judgement relies on a complex interaction of emotional mechanisms with cognitive ones (Greene, Sommerville, Nystrom, Darley, & Cohen, 2001) inducing a positive emotion before making judgements on moral dilemmas increasing the number of utilitarian responses people make (Valdesolo & DeSteno, 2006). Utilitarian choices in moral dilemmas are usually characterized by judgements of preferring the collective welfare over the welfare of fewer individuals, and would rely more on cognitive reasoning rather than emotional processes.

It is argued that utilitarian choices in difficult moral dilemmas involve cognitive control processes located in the Prefrontal Cortex (PFC). Results from functional Magnetic Resonance Imaging (fMRI) and lesion studies indicate that the PFC is essential for higher cognitive functions including regulating one’s emotions and solving complex social dilemmas (Forbes & Grafman, 2010). More specifically, it is suggested that judging moral dilemmas activate a network of brain regions including the ventrolateral PFC, and the medial frontal gyrus (Moll, Eslinger, & de Oliveira-Souza, 2001; Moll, de Oliveira-Souza, & Zahn, 2008).

Although there is evidence for a relationship between cognition, emotion, and moral judgement (Greene et al., 2001; Greene, Nystrom, Engell, Darley, & Cohen, 2004), and the involvement of PFC in the aforementioned functions, there is still a limited understanding of how cognitive and emotional processes subserve moral judgements. Moreover, there is no conclusive data concerning the role of the most anterior part of the prefrontal cortex (the Frontopolar Cortex; FPC) in higher cognitive functions. A number of functional neuroimaging studies reported the FPC activation during the performance of complex mental tasks (Cabeza & Nyberg, 2000).

Although functional neuroimaging studies provided much evidence for the role of PFC in moral judgement, it cannot be definitively established that a specific brain area is necessary for a particular cognitive process. Therefore, the current study investigated six patients with lesions in the right FPC and 10 healthy individuals in personal and impersonal moral dilemmas before and after inducing negative emotions. Patients and controls also performed tests of executive control. The study was also aimed at replicating previous observation of changes in moral judgement following emotion induction (Valdesolo & DeSteno, 2006). Also, with regards to group differences, the study aimed at examining the causal role of the right FPC in moral judgement, and the causal role of the FPC on the way in which emotion affects moral judgment.

The study also hypothesized that if the rFPC involves emotional processing, rFPC damage may lead to an increased number of utilitarian responses to moral dilemmas, since the affected individuals may rely more heavily on cognitive operations to make moral judgements. Patients may thus be relatively unaffected by the manipulation of inducing emotions prior to making moral judgements. Conversely, if the rFPC plays a role primarily in cognitive control functions, it is expected that damage leads to a reduced number of utilitarian responses since they may rely predominantly on emotional processes to make moral judgements. Finally, patients might be highly affected by emotion induction given their reduced ability to regulate emotions and utilize cognitive processes for judging moral dilemmas.

## 2. Methods

Participants: Seven patients with brain damage to the FPC area were recruited from hospitals affiliated to the University of Tehran (Imam Khomeini and Sina) as well as MRI centers (Athari, Imam Khomeini, and Jam-e-Jam). One of the patients was excluded since the damage was in the left FPC. Thus, six patients (four females; aged 32–64 years) with rFPC lesions were enrolled in the study. The study protocol was approved by the local Ethics Committee. All patients signed written informed consent prior to participation in the study. Patients were included in the study based on the following criteria: (a) age above 18 at the time of brain damage; (b) focal and stable brain damage in the right FPC; (c) at least one year passed from operation; and (d) absence of other neurological and psychiatric disorders, or substance abuse. Also, patients showed no decline in social interpersonal conducts according to reports from family members.

**Figure 1.**
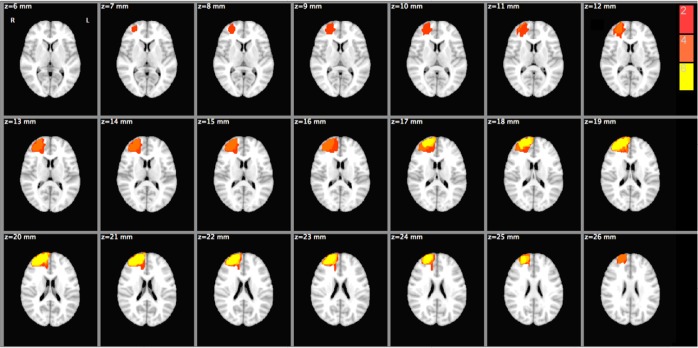
The neuroanatomical analysis of the brain lesions among six patients with the rFPC damage Representation of the damage to rFPC using MRI; The yellow color indicates the overlap between six patients, orange color represents overlap of the damage among four patients, and red color indicates overlap between damage areas among two patients.

All patients showed normal self-awareness, and all except one, could manage independent life (e.g. maintain their personal cleanliness, carrying out functions such as cooking and going to work). Three patients had penetrating head injury and three others had histories of excised brain tumors. The location of the lesions was confirmed by MRI scans (Figure 1). Ten healthy volunteers matched by age and educational level were also recruited. Exclusion criteria for healthy controls were having epilepsy, brain damage or other neurological conditions, and psychiatric problems such as depression. Demographic characteristics of the study groups are shown in Table 1.

**Table 1.** Demographic data and IQ test

**Demographics**	**Patients**	**Normal**
Gender M:F	2:4	4:6
Age, yr	46.3±11.7	43.1±9.4
Education, yr	12.5±2.3	14.8±1.6
Chronicity, yr	10.2±7.8	n/a
IQ	115.7±9.4	117.0±10.6

Note: Chronicity=Time since the brain damage occurred in years. Abbreviations: F=Female; M=Male; rFPC=patients with right Frontopolar Cortex lesion; IQ, Wechsler Adult Intelligence Scale-III-Persian version (WAIS-III) full-scale IQ.

**Table 2.** The mean of RTs and responses in MJ between the control and the rFPC groups

**Measures**	**Control**						

	**Personal**			**Impersonal**						
	
	**MJ1**	**MJ2**	**MJ3**	**MJ1**	**MJ2**	**MJ3**
Mean±SD	7.88±5.31	3.38±4.37	6.74±4.73	6.98±7.34	4.61±5.45	6.50±2.8
Responses	0.45±0.28	0.20±0.31	0.42±0.31	8	9	9

**Measures**	**rFPC**						

	**Personal**			**Impersonal**						
	
	**MJ1**	**MJ2**	**MJ3**	**MJ1**	**MJ2**	**MJ3**
Mean±SD	6.48±3.33	6.8±3.40	6.93±5.40	6.04±4.79	6.32±5.79	5.58±2.76
Responses	0.33±0.26	0.29±0.19	0.5±0.31	4	4	6

Note: The proportion of utilitarian responses in personal dilemmas and the number of appropriate responses in impersonal.

RTs: Response Times; MJ: Moral Judgment

### 2.1. Structural imaging

All patients underwent structural brain MRI. Structural MR images were linearly registered to the MNI template, and the area of brain damage was manually outlined on each image. A mask outlining the lesion was made for each patient and was overlaid on the MNI template in order to provide a population map. The neuro-anatomical analysis showed that the brain lesions were mainly located in the rFPC as illustrated in Figure 1.

### 2.2. Neuropsychological assessment and behavioral tasks

All participants underwent neuropsychological assessment including the Persian version of the Wisconsin Card Sorting Test (WCST), the Wechsler Adult Intelligence Scale-III (WAIS), and the Color-Word (C-W) Stroop Test.

In order to assess moral judgement, participants made judgments on a series of hypothetical scenarios that involved a moral dilemma. This task was adapted from a previously published study (Greene et al., 2001). It is argued that some moral dilemmas, referred to as “personal” are primarily driven by socioemotional processes, while other moral dilemmas, referred to as impersonal, are mainly associated with cognitive processes (Greene et al., 2001; 2004). In personal moral dilemmas, people are often faced with the following: in order to maximize aggregate welfare (for example saving the lives of several individuals) one should commit a personal moral violation (killing ones’ own child).

According to the current theory (e.g. Greene et al., 2004) this dilemma is particularly difficult, since the negative socio-emotional response associated with the thought of killing individual`s own child interferes with the rational of committing this act for the sake of the group. All moral dilemmas were directly translated from English into Persian except three, which were considered culture-bound and therefore excluded.

The moral dilemmas were evaluated in a separate study on healthy volunteers (Ziaei, Khodapanahi, Heidari, & Keshvari, 2009) to assess the level of conflict for each dilemma (high or low) according to previously published criteria (Koenigs et al., 2007). Participants listened to the audio of the scenario with an average speed of 2.6 words per second and then a question about a hypothetical action related to the scenario was presented on a 15 inch LCD monitor. The participants’ responses were collected within a 45 second time frame after the question to make sure they had enough time to decide. Their responses were collected by asking participants to press the right Shift key for a “yes” response and the left Shift key for a “no” response.

Based on previous observations indicating that emotional state affects moral judgement in general, and personal dilemmas in particular (Wheatley and Haidt, 2005; Schnall et al., 2008; Valdesolo & Desteno, 2006), negative feelings were induced by presenting emotional stimuli prior to participants moral judgements. By inducing a feeling of negativity it was anticipated that participants would automatically utilize emotion regulation to cope with their feelings. This would thus reduce the perceived negativity on subsequent moral violations, and thereby increase the number of utilitarian responses, which presumably are relying more heavily on cognitive rather than emotional involvement.

**Figure 2.**
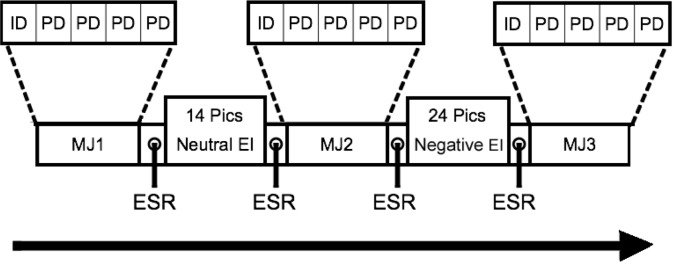
Experimental design Three blocks of moral judgments were presented (MJ1–MJ3). Within each moral judgment blocks, four Personal (PD) and one Impersonal Dilemmas (ID) were presented. Two emotional manipulation steps were also embedded in the designs that were either neutral or negative, referring to Emotion Induction (EI). Prior to and following each emotion induction, participants were asked to indicate their emotional states, referring to Emotional Self-Report (ESR).

Thus, the entire computerized task consisted of overall three blocks of Moral Judgment (MJ) and two blocks of Emotion Induction (EI). In total, 12 moral dilemmas were included. In each block of moral judgement (MJ1 to MJ3), five dilemmas (four personal and one impersonal) were presented. Moral judgment blocks were presented either (i) without presentation of any pictures (MJ1 or baseline), (ii) after presentation of neutral pictures (MJ2), and (iii) after presentation of negatively salient emotional pictures (MJ3). The emotion induction block consisted of either neutral (for MJ2) or negative (for MJ3) blocks of pictures presented before moral dilemmas (Figure 2).

The emotion induction task consisted of 14 neutral and 24 negative emotional pictures from the International Affective Picture System (IAPS; Lang Bradley, & Cuthbert, 1995). Negative pictures portrayed moral violations; e.g. physical assaults, child physical abuse, and war scenes (Haidt, 2003) with the mean valence of 6.12±0.88, and each picture was displayed for five seconds. In addition, the Positive Affect Negative Affect Schedule (PANAS; Watson Clark, & Tellegen, 1988) was employed, in which participants were asked to rate their emotional experience (self-report for excitement, anxiety, guilt, anger, and sadness) before and after each block of neutral and emotional pictures on a scale from 0–7 (0=not at all, 7=very much). Before the initiation of the experiment, all participants were instructed on how to perform the tasks, and were also given practice runs.

### 2.3. Data analysis

The study employed repeated-measures ANOVA to assess group differences across the three blocks of moral judgment to examine the effect of rFPC lesion on Response Times (RTs) and the proportion of their utilitarian/appropriate responses. Also, intergroup differences in performance for the three blocks of MJ were tested separately for personal and impersonal dilemmas. Finally, the emotional self-reports before and after each stimulus was analyzed to ensure that the stimulus had the expected emotional effect using repeated measures ANOVA. Separate analyses using two-sample t-test between control and patient groups were performed for tests on executive control functions using the C-W Stroop test and WCST.

## 3. Results

Demographic data showed no significant differences in age (t (14)=1.17, P=0.261; Table 1) as well as general intelligence using WAIS-III (t (14)=1.031, P>0.05). However, the number of years of education was lower among the patients than controls (t (14)=3.34, P=0.02; Table 1). To estimate the effects of subjective experience by negative emotion induction, ratings of each participant’s emotional self-report before and after inducing negative emotions were used. Repeated measures ANOVA showed that for all types of emotions, participants reported a more negative emotional state after viewing the negative emotional pictures compared with their self-ratings prior to negative emotion induction (F (3,42)=37.35, P<0.001). Across emotional ratings, no differences were observed between patients and controls (F (1,14)=2.33, P=0.149), suggesting that groups did not differ significantly in the effect of negative EI.

**Figure 3.**
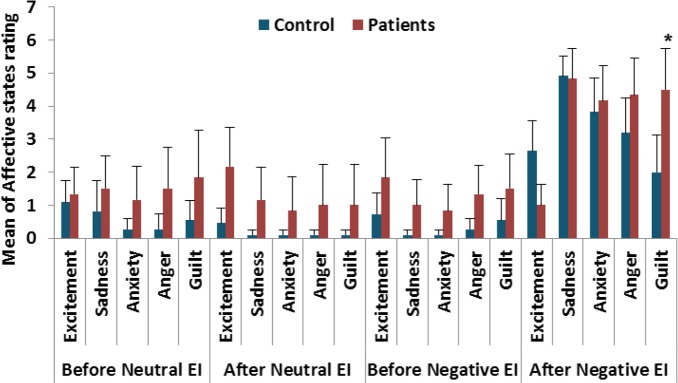
Emotional self-report Prior to and following Emotion Induction (EI) steps, participants reported their emotional states in five areas, excitement, anxiety, guilt, anger, and sadness. The results showed that patients significantly rated their guilt responses higher than those of the control group after negative emotion induction, indicated by asterisk.

When specific emotional states were considered, it was observed that after negative EI, patients reported stronger feelings of guilt, compared with the control group (F (1,14)=6.9, P=0.020; Figure 3). Possibly, there might be an association between feelings of guilt after emotion induction and the rFPC. With regards to moral judgement, two measures were used; the time it took to solve the dilemma (RT) and the number of appropriate responses. For consistency, the term utilitarian responses was used for ‘yes’ responses to personal dilemmas and appropriate responses for ‘yes’ responses to impersonal dilemmas. The proportion of utilitarian responses, along with the RTs, for personal dilemmas and frequencies of appropriate responses for impersonal dilemmas are shown in Table 2.

First, it was assessed whether there was a group difference in participants’ responses to moral judgements. It was analyzed whether there were any baseline group differences in the first block of moral judgment assessment in which neither neutral nor negative emotional pictures were presented. A intergroup ANOVA on the proportion of utilitarian responses showed no significant differences between patients with rFPC damage and controls (F (1,14)=0.675, P=0.425). This suggested that damage to the rFPC does not have a general impact on the proportion of utilitarian responses. Second, in order to assess differences in the proportion of appropriate choices, Fisher exact test (e.g. Agresti, 2001) was employed for statistical inference with small sample sizes and low frequency data. The results of the Fisher exact test showed that the response to impersonal dilemmas was not significantly different between the two groups (P>0.5). These results suggested that damage to rPFC did not affect how patients responded to either personal or impersonal dilemmas.

Next, to examine whether there were any differences between the two groups in RTs of moral judgments before negative EI, the performances in MJ1were compared. The results showed no significant differences between patients with rFPC damage and controls either for personal dilemmas (F(1,14)=0.333, P=0.573) or impersonal dilemmas (F(1,14)=0.077, P=0.785).

In order to assess the impact on right FPC lesions on moral judgement after emotion induction, first the RTs of appropriate responses in moral judgment blocks in patients and controls were compared using a group (patient vs. control)×types of dilemmas (personal vs. impersonal)×emotion induction (baseline vs. neutral vs. negative) repeated measures ANOVA. The study was particularly interested in testing the hypothesis of whether emotion induction prior to moral judgement would have a specific effect on ‘yes’ responses among patients with rFPC damage compared with controls.

The results showed no significant main effect of group [F(1,14)=0.051, P=0.824], emotion induction [F(2,28)=0.862; P=0.433] or type of dilemma [F(1,14)=0.41; P=0.532] on the time it took to make a moral judgement. In order to test the hypothesis that patients would be affected in making utilitarian choices in moral judgements following negative emotion induction, the three-way interaction between group, type of dilemma, and emotion induction was examined. This interaction was not significant [F(2,28)=0.118, P=0.889], suggesting no significant differences between the performance of the two groups in responding to moral judgments prior and following negative emotion induction. None of the other interactions were significant.

In addition to analyses of RT, the effect of emotion induction on the number of ‘yes’ responses before and after emotion induction among the two groups were investigated. Similar to the RT analyses, the repeated measures (patient vs. control)×emotion induction (baseline vs. neutral vs. negative) ANOVA on the proportion of utilitarian responses of personal dilemmas was employed. No significant main effect of group [F(1,14)=0.018; P=0.896] was observed, suggesting a lack of general deficits in patients with rFPC damage on moral judgement responses.

A significant main effect of emotion induction on the utilitarian responses was observed [F(2,28)=5.257; P=0.012] showing that the number of utilitarian responses increased following negative rather than neutral emotion induction. This suggested that the induction of negative emotion increased the number of utilitarian responses to personal dilemmas. Furthermore, with regards to impersonal dilemmas the results showed no significant differences between appropriate responses in the baseline relative to after neutral (P=0.736) or negative emotion induction (P=0.538), suggesting that responses to impersonal dilemmas were not affected by emotion induction.

**Figure 4.**
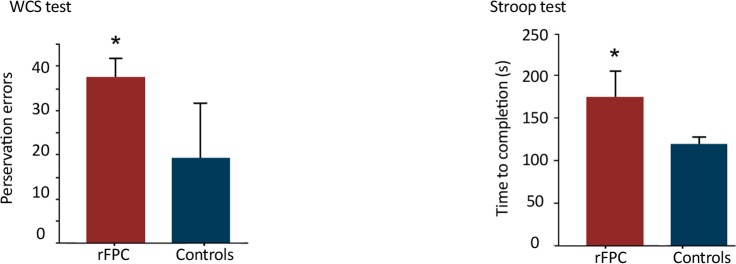
Performance on executive control tasks patients with rFPC damage The results showed more preservation errors in Wisconsin Card Sorting (WCS) test relative to healthy controls. Furthermore, patients spent more time to complete Stroop test relative to controls rFPC=right frontopolar cortex.

In order to test the effect of right FPC lesions on executive control, two measures from the WCST were used. First, using a one-tailed t-test it was observed that the mean perseveration errors significantly increased in the patients compared with controls [t(14)=1.93, P<0.05[. Second, the number of completed categories significantly reduced in the patients [t(14)=3.24, P<0.05]. For the Stroop test, a similar pattern of reduced performance was observed in patients showing longer mean response time to complete the task [t(14)=2.90, P<0.05]. However, there were no significant intergroup differences in the number of errors in this test. The obtained results suggested reduced response inhibition/task-set maintenance ability in patients with right FPC lesions (Figure 4).

## 4. Discussion

The current study explored the role of rFPC in moral judgement, its role in mediating emotional processes when responding to moral dilemmas, and its function in executive control. First, it was observed that inducing an emotional state increased the number of utilitarian responses both in patients and controls. Second, no significant differences were observed in response time or the number of utilitarian responses in the patients compared with controls suggesting that rFPC damage may not directly cause impairments in moral judgement. Third, no significant intergroup differences were observed in personal and impersonal dilemmas prior to and following emotion induction, suggesting that rFPC was not directly mediating the effect of emotion on moral judgment. Finally, performance on Stroop test inhibition and WCST was impaired in patients compared to controls suggesting that rFPC was critically involved in executive control processes required for these tests.

The current study findings were in line with those of the previous studies demonstrating that manipulations of emotion can alter moral judgment (Wheatley & Haidt, 2005; Schnall, Benton, & Harvey, 2008; Valdesolo & Desteno, 2006; Strohminger, Lewis, & Meyer, 2011). For example, Valdesolo and DeSteno showed that inducing positive emotion by showing a comedy clip increased the odds of selecting a utilitarian response in a personal dilemma; they argued that positive emotions reduced the negative feeling that would normally accompany a utilitarian response in a personal dilemma.

The current study findings showed that negative emotion induction had a similar effect by increasing the number of utilitarian responses in personal dilemmas. While it seems contradictory to the findings by Valsesolo and DeSteno, one possibility is that when participants are viewing negative emotional pictures they are also engaging emotion regulation processes to cope with the negative feelings elicited by such pictures. Speculatively, subsequent moral judgements may therefore be experienced as less negative given the activation of emotion regulation processes leading to an increased number of cognitively driven utilitarian responses. The current study findings soundly corroborated previous observations suggesting that emotional influences from the environment can alter the emotional state of an individual and higher cognitive functions such as reasoning and moral judgment.

While previous findings suggested a specific role for the right PFC in moral judgement (Moll et al., 2002), the current study observations suggested no group differences in general performance accuracy on moral judgements. For example, previous studies showed that the right anterior PFC (BA 10) is activated for utilitarian responses in high conflict dilemmas (Greene et al., 2004). Yet other studies showed that damage to vmPFC increased the number of appropriate utilitarian responses while at the same time shortening the RTs to make such decisions (Ciaramelli, Muccioli, Làdavas, & Pellegrino, 2007; Koenigs et al., 2007; Mendez, 2006). These results suggested a reduced emotional influence on moral dilemmas following damage to regions known to be involved in emotional processing. The current study finding that the number of utilitarian responses is not critically affected by rFPC damage suggests that regions other than rFPC are more directly involved in emotional processing affecting moral judgement. Therefore, the rFPC is not solely responsible for emotional influences on moral judgment. Also, there were no significant differences of moral judgments (RTs and responses) between the two groups following negative emotion induction. This observation, together with the finding of an increased number of utilitarian responses following emotion induction across both groups, suggests that rFPC may not have a direct role in mediating emotional processes during moral judgments.

Interestingly, it seems that how guilt is felt might be affected by damage to the rFPC. Although the feeling of guilt did not have an impact on moral responses, patients with rFPC damage reported elevated feelings of guilt following negative emotion induction compared to controls. While the current study aimed at investigating whether negative emotions influence moral judgments in patients with rFPC damage and controls, it is important to emphasize that negative emotion is a broad term that incorporates many different emotions (for a discussion, see Haidt, 2003). This finding was in accordance with those of the other studies concerning brain areas involved in moral emotions (Moll et al., 2002).

These results provided evidence for the role of rFPC in specific executive control processes such as inhibition and set-shifting measured by the Stroop test and WCST, respectively. Reduced performance in tasks requiring inhibition in patients with right PFC damage is in accordance with previous observations of reduced response inhibition ability, assessed by means of stop-signal and go/no-go task performance (Aron, Robbins, & Poldrack, 2004; Aron, Fletcher, Bullmore, Sahakin, & Robbins, 2003; Clark et al., 2007). However, the view of inhibitory control as mainly associated with right PFC is challenged by previous observations of divergent neuropsychological evidence obtained in studies that did not observe stop-signal or go/no-go deficits in patients with right PFC damage (see Swick, Ashley, & Turken, 2008 for a review). Also, there is limited evidence for a stronger involvement in right compared to the left PFC in the Stroop test (Alvarez & Emory, 2006). Therefore, the role of frontal regions with a specific involvement of the right PFC in inhibitory control still remains unanswered.

The current study results provided additional evidence for the role of the right PFC in general, and the right FPC in particular in inhibitory control functions. These findings also corroborate recent observations from functional imaging studies (eg Lenartowicz, Verbruggen, Logan, & Poldrack, 2011; Firstmann et al., 2008; Rubia, Smith, Brammer, & Taylor, 2003; Brench, 1993; Carter, Mintun, & Cohen, 1995; Roberts & Hall, 2008) showing robust activation in right, and also left, PFC regions during response inhibition tasks. However, activation is primarily localized in the inferior part of the PFC, rather than the frontopolar cortex. Thus, the current study results suggested that the rFPC plays an important role in inhibition, possibly as one key area within a set of regions important for executive control.

The dissociation showing impaired inhibitory ability following rFPC damage while leaving performance on moral judgment intact suggests that moral judgment may critically depend more heavily on emotional perception rather than cognitive inhibitory functions. Additionally, this finding again supports the role of other areas rather than only rFPC in moral judgment. Also, the current study findings of reduced WCST performance in FPC patients compared to those of controls was in line with evidence suggesting impaired performance on the WCST in patients with PFC lesions. Most of the current studies support the sensitivity of WCST to frontal lobe lesion (Alvarez and Emory, 2006), but it is unclear whether there is a specific role of the right PFC in WCST. In fact, while some studies reported no difference in the side of damage to the frontal cortex (Goldstein, Obrzut, & John, 2004), others reported no difference in laterality of damage in the frontal cortex (Demakis, 2003).

While the current study results were in line with the findings of WCST deficits in frontal patients in general, it is evident from previous lesion studies showing reduced performance in WCST following damage in various parts of the frontal cortex that performance in this task is not specifically associated with the right FPC. Possibly, the right FPC is one important region in a network of frontal sites supporting executive control functions such as the WCST.

There were a few limitations to this study that need to be emphasized. First, the difficulty of recruiting participants with lesions to the FPC resulted in only a small sample size of patients. A lack of comparison with patients with other brain damage, together with the small sample size possibly limits the conclusions that can be drawn about the role of right FPC on the emotional influence on moral judgements and executive control. However, it should be noted that the increasing number of utilitarian responses in patients with ventromedial prefrontal lesions was previously tested with equivalently small sample sizes (Koenigs et al., 2007; Ciaramelli et al., 2007). Another potential reason shared with most lesion studies is that the lack of an effect of rFPC lesion on moral judgement could be that the brain’s plasticity diluted subsequent behavioral effects by compensation in homologous or surrounding brain tissue.

The current study results showed that inducing an emotional state increased the number of utilitarian responses both in patients and controls. No significant differences were observed in response time or number of utilitarian responses in patients compared to controls suggesting that rFPC damage may not directly cause impairments in moral judgment. Also, no significant intergroup differences were observed in personal and impersonal dilemmas prior to and following emotion induction, implicating that rFPC is not directly mediating the effect of emotion on moral judgment. Finally, the results from executive control functions showed reduced performance in patients with rFPC damage compared to controls.

## Ethical Considerations

### Compliance with ethical guidelines

The study was approved by the Ethics Committee at School of Psychology, Shahid Beheshti University.
